# Assessment of Extreme Risk Protection Order Use in California From 2016 to 2019

**DOI:** 10.1001/jamanetworkopen.2020.7735

**Published:** 2020-06-18

**Authors:** Rocco Pallin, Julia P. Schleimer, Veronica A. Pear, Garen J. Wintemute

**Affiliations:** 1 Violence Prevention Research Program, Department of Emergency Medicine, University of California Davis School of Medicine, Sacramento

## Abstract

**Importance:**

A total of 19 states and the District of Columbia now have extreme risk protection order (ERPO) or similar policies, and others are considering them; however, little research exists describing their use.

**Objective:**

To characterize early use of California’s ERPO policy by providing the first aggregate, statewide description of ERPOs, individuals subject to them, and petitioners.

**Design, Setting, and Participants:**

This cross-sectional study analyzed 1076 respondents to ERPOs recorded in the California Department of Justice California Restraining and Protective Order System from 2016 to 2019. Descriptive analyses of orders issued between January 1, 2016, and December 31, 2019 in California were performed, and univariate Moran I was calculated to examine county-level spatial autocorrelation of the policy’s use.

**Main Outcomes and Measures:**

The primary study outcomes included the characteristics of ERPO respondents (demographic characteristics), petitioners (law enforcement vs family or household members), and orders (type and service) as well as temporal and spatial variation in policy use during the first 4 years of implementation.

**Results:**

Of 1076 respondents during the study period, most were men (985 [91.5%]) and white individuals (637 [59.2%]), with a mean age of 41.8 years (range, 14 to 98 years). A law enforcement officer was the petitioner in 1038 cases (96.5%). The number of respondents increased during the study period from 70 in 2016 to 700 in 2019, and there was substantial county-level variation in ERPO use (ranging from 0 to 354 respondents), with significant spatial clustering in counts of ERPO respondents among neighboring counties (observed Moran I, 0.18, mean [SD] Moran I from reference distribution, −0.01 (0.05); *z* value, 3.58; *P* = .004).

**Conclusions and Relevance:**

This study, among the first to describe the early utilization of an ERPO or similar policy, found a substantial increase in the use of ERPOs in California from 2016 to 2019. These results could inform policy makers and other stakeholders involved in policy implementation and outreach in California and elsewhere. Similar studies in other states would be useful to understand variation.

## Key Points

**Question:**

What were the characteristics of extreme risk protection order (ERPO) petitioners and respondents, and what were the spatial and temporal patterns in the policy’s use in California during the first 4 years of its implementation?

**Findings:**

This cross-sectional study of 1076 ERPO respondents found that most respondents were men and white individuals and that most petitioners were law enforcement officers. Use of ERPOs increased substantially from 2016 to 2019, and there was large variation in uptake between counties.

**Meaning:**

The results of this study could inform stakeholders involved with ERPO policy adoption, implementation, education, and outreach as well as researchers seeking to further evaluate these policies.

## Teaser

This cross-sectional study characterizes the early use of California’s extreme risk protection order (ERPO) policy by providing an aggregate, statewide description of ERPOs, the individuals subject to them, and petitioners.

## Introduction

Firearm injury and death are major public health problems in the US. In 2018, there were 39 740 deaths from firearms, 24 432 (61.5%) from suicide and 13 958 (35.1%) from homicide.^1^ That year, 51% of all suicides and nearly 75% of all homicides involved firearms.^1^ In California, where firearm deaths result nearly equally from homicide and suicide, there were 3040 such deaths in 2018.^1^ Homicide and suicide rates in California vary significantly at the county level.^2^

Many firearm injury prevention strategies target members of groups considered to be at increased risk of harming themselves or others. For example, federal law prohibits firearm purchase and possession for those convicted of a felony or a domestic violence misdemeanor, subject to a domestic violence restraining order, designated an “unlawful user of or addicted to any controlled substance,” “adjudicated as a mental defective or … committed to any mental institution,” and those who have renounced their US citizenship as well as fugitives and persons “unlawfully in the United States.”^3^ Some state laws place prohibitions on additional groups. However, many who harm themselves or others with firearms are not prohibited persons.^3,4^

One mass shooting in California demonstrated how the existing law failed to prohibit certain dangerous individuals from possessing firearms and provided legislative momentum for California’s extreme risk protection order (ERPO) policy. In April 2014, Santa Barbara County sheriff’s deputies conducted a welfare check in response to a call from the concerned parents of Elliot Rodger, aged 22 years and living in Isla Vista, California, who was not prohibited from owning firearms. The deputies lacked probable cause to arrest Rodger, to put him on an involuntary mental health hold, or to search his apartment, and they did not check state records of firearm purchases or view the concerning online videos mentioned by Rodger’s mother.^5^ Three weeks later, Rodger killed 6 and injured 14 using a combination of firearms, stabbing, and vehicle ramming in a self-proclaimed act of retribution. Shortly thereafter, California adopted an ERPO law, similar to existing risk warrant laws in Connecticut and Indiana.^6^

In California, ERPOs are called gun violence restraining orders (GVROs). Since January 1, 2016, the GVRO policy has allowed for temporary removal of firearms and ammunition from individuals who pose a risk of violence to themselves or others but who are not otherwise prohibited from possessing firearms and ammunition and for whom other risk reduction interventions (eg, arrest, other protective orders, emergency psychiatric hospitalizations) are not available or have failed.^6^ Individuals subject to GVROs are also prohibited from purchasing firearms and ammunition for the duration of the order.

California has 3 types of GVROs, as follows: emergency orders, temporary orders (hereafter referred to as ex parte orders), and orders issued after notice and hearing. Emergency orders are available to law enforcement 24 hours a day for rapid response in crisis situations. Family members, household members, and law enforcement may petition for ex parte orders and orders issued after notice and hearing. Emergency and ex parte orders last up to 3 weeks; orders issued after notice and hearing last up to 1 year. Standards of evidence vary by type of order, but all are issued by a judge or magistrate who reviews the case for evidence of present risk. All types of GVROs are renewable. On an order’s expiration or termination, the person subject to the order (hereafter referred to as the respondent) can ask the court to return his or her firearm(s) and ammunition, triggering a new background check. If the respondent is not prohibited for other reasons, the recovered firearms are returned.^6^

Although California’s ERPO law grew out of a mass shooting event, ERPOs also have promise for firearm suicide prevention. Most deaths from firearms in the US are suicides, and firearms are the most lethal means of suicide.^7^ ERPOs provide a legal tool for reducing access to lethal means among people at imminent risk of self-harm. Although, to our knowledge, there is very limited research on ERPO use and effectiveness, studies of similar risk warrant policies suggest that extreme risk laws are effective for suicide prevention. Evidence from Connecticut and Indiana, where risk warrant legislation has existed since 1999 and 2005, respectively, suggests that such laws have a number needed to treat of 10 to 20 (in this case, the number of firearm removal actions needed to prevent 1 suicide).^8,9,10^

Our 2019 analysis of a subset of California GVRO cases^11^ found that such laws may also be useful for preventing mass shooting events. That analysis reported 21 cases in which orders were sought with the intent to prevent mass violence.^11^ No mass violence occurred in those cases, but such data provide only limited support for a claim that ERPOs directly prevent mass violence.

Publicized cases have demonstrated the breadth of GVRO use, including prevention of interpersonal violence, suicides, and threatened mass shootings. For example, in a suburb of Sacramento, police petitioned for a GVRO in response to a call from a concerned relative and recovered firearms from a man threatening to commit suicide.^12^ In San Diego, a GVRO was granted for a man with dementia who made threats to shoot his wife and neighbor, believing they were having an affair.^12^ In another San Diego case, a GVRO was used to recover a semiautomatic rifle with “significant killing capability” from a man who praised a recent mass shooter and made threats to bring his gun to work.^13^

As of May 2020, 19 states and the District of Columbia have ERPO or similar risk warrant statutes. A better understanding of California’s experience may inform policy and practice in California and elsewhere. This study describes California’s GVRO respondents and petitioners as well as the temporal and geographic variation in the policy’s use during its first 4 years.

## Methods

### Data Sources

Data on GVROs were obtained from the California Department of Justice (CA DOJ) California Restraining and Protective Order System (CARPOS) for the period January 1, 2016, to December 31, 2019. CARPOS is a case-finding tool that maintains information on respondent demographic information, including age, gender, and race/ethnicity (CARPOS categories, which may be based on petitioner classification or respondent self-report, were collapsed into white, black, Latinx, Asian, and other or unknown), order and petitioner types, and order service (ie, whether the order was physically delivered directly to the respondent, thereby putting the order into effect). eTable 1 in the Supplement provides a complete list of variables available in CARPOS. Information is entered into CARPOS by law enforcement officers or court personnel. Although respondents often have more than 1 order, CARPOS only retains the most recent order, so analyses only include each respondent’s most recent order from any year from 2016 to 2019. The University of California Davis institutional review board approved this study and waived informed consent because the study was deemed to have minimal risk. This report follows the Strengthening the Reporting of Observational Studies in Epidemiology (STROBE) reporting guideline for cross-sectional studies.

### Statistical Analysis

We determined counts and percentages of GVROs and characteristics of respondents and petitioners from 2016 to 2019. Because we presented data for the entire population of respondents, rather than a sample, measures of error, such as confidence intervals, were not calculated. We mapped counts and rates of respondents per county from 2016 to 2019 and analyzed geographic clustering with spatial data analysis.^14^ To examine spatial dependence, we calculated univariate Moran I using a first-order Queen contiguity matrix to define neighboring counties (ie, counties are considered neighbors if they share a boundary). We used a permutation test, using 999 random permutations, to determine statistically significant spatial autocorrelation (at 2-tailed α = .05). Significant positive spatial autocorrelation for GVRO counts (or rates) would mean that neighboring counties have issued a similarly high or low number of GVROs (or GVROs per population), indicating that either the use of GVROs in 1 county increases the use of GVROs in neighboring counties or that some exogenous factor that is also spatially clustered is contributing to GVRO use. Analyses were conducted using Stata SE version 15.1 (StataCorp), QGIS version 2.18.28, and GeoDa version 1.6.7.

## Results

**Figure 1.**
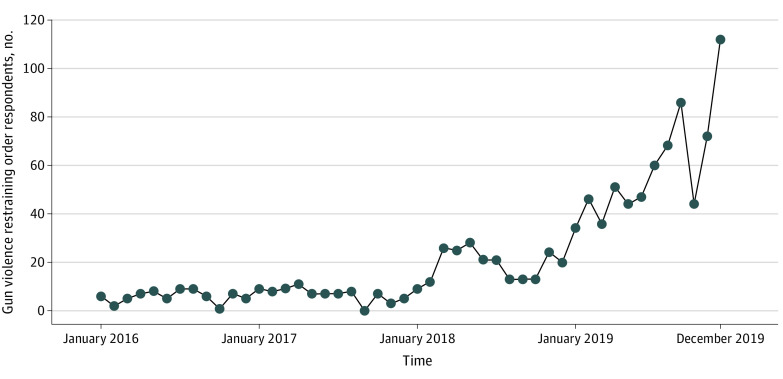
Counts of Gun Violence Restraining Order Respondents by Month, 2016 to 2019

There were 1076 respondents to GVROs during 2016 to 2019. Of these, 985 (91.5%) were men and 637 (59.2%) were white individuals (Table). The racial/ethnic distribution of GVRO respondents roughly matches that of firearm owners in California (ie, 64% white, 20% Latinx, 4% black, and 9% other race/ethnicity)^15^; however, compared with the state as a whole (ie, 38% white, 39% Latinx, 6% black, and 14% Asian),^16^ a larger percentage of GVRO respondents were white individuals and smaller percentages were Latinx or Asian individuals. The mean age of respondents was 41.8 years (range, 14 to 98 years) (Table). Law enforcement officers petitioned for 1038 of 1076 orders (96.5%). The annual number of respondents increased by 900%, from 70 in 2016 to 700 in 2019, with a dramatic increase from 2018 to 2019 (225 vs 700) (Figure 1). San Diego County had 262 more respondents in 2019 than in 2016 (267 vs 5) and accounted for nearly 40% of the total increase. The counties with the next highest absolute increases were Orange and Santa Clara Counties, with 59 (62 vs 3) and 49 (55 vs 6) more respondents in 2019 than in 2016, respectively.

**Table.** Characteristics of Gun Violence Restraining Order Respondents, From the California Restraining and Protective Order System, 2016 to 2019

Characteristic	No. (%)^a^				
	2016 (n = 70)	2017 (n = 81)	2018 (n = 225)	2019 (n = 700)	Total (N = 1076)
Age					
<18	0	2 (2.5)	6 (2.7)	13 (1.9)	21 (2.0)
18-29	13 (18.6)	16 (19.8)	62 (27.6)	158 (22.6)	249 (23.1)
30-44	21 (30.0)	24 (29.6)	73 (32.4)	262 (37.4)	380 (35.3)
45-59	24 (34.3)	21 (25.9)	53 (23.6)	173 (24.7)	271 (25.2)
≥60	11 (15.7)	18 (22.2)	31 (13.8)	94 (13.4)	154 (14.3)
Gender					
Women	14 (20.0)	4 (4.9)	12 (5.3)	61 (8.7)	91 (8.5)
Men	56 (80.0)	77 (95.1)	213 (94.7)	639 (91.3)	985 (91.5)
Race/ethnicity					
White	46 (65.7)	54 (66.7)	132 (58.7)	405 (57.9)	637 (59.2)
Black	4 (5.7)	2 (2.5)	17 (7.6)	70 (10.0)	93 (8.6)
Latinx	9 (12.9)	10 (12.3)	55 (24.4)	147 (21.0)	221 (20.5)
Asian	4 (5.7)	8 (9.9)	13 (5.8)	47 (6.7)	72 (6.7)
Other or unknown	7 (10.0)	7 (8.6)	8 (3.6)	31 (4.4)	53 (4.9)
Petitioner					
Law enforcement	62 (88.6)	75 (92.6)	221 (98.2)	680 (97.1)	1038 (96.5)
Family or household member	6 (8.6)	6 (7.4)	4 (1.8)	20 (2.9)	36 (3.5)
Order type					
Emergency	47 (67.1)	65 (80.2)	112 (49.8)	223 (31.9)	447 (41.5)
Ex parte	16 (22.9)	9 (11.1)	36 (16.0)	239 (34.1)	300 (27.9)
Order issued after notice and hearing	7 (10.0)	7 (8.6)	77 (34.2)	238 (34.0)	329 (30.6)
Order served					
Yes	61 (87.1)	71 (87.7)	170 (75.6)	410 (58.6)	712 (66.2)
No	9 (12.9)	10 (12.3)	55 (24.4)	290 (41.4)	364 (33.8)

^a^ Two orders had missing petitioner types in 2016; 1 respondent had missing birthdate. All data are displayed for the last order per person during the entire study period (2016-2019).

A total of 712 orders (66.2%) were served; rates of order service were stable from 2016 to 2017, but declined by 13.8% from 2017 to 2018 (71 [87.7%] vs 170 [75.6%]) and by 22.5% from 2018 to 2019 (170 [75.6%] vs 410 [58.6%]). With San Diego cases excluded, the proportion of orders served remained relatively stable until 2019 (57 of 65 [87.7%] in 2016; 66 of 74 [89.2%] in 2017; 128 of 150 [85.3%] in 2018; 321 of 433 [74.1%] in 2019). Information on reasons for nonservice was not available.

**Figure 2.**
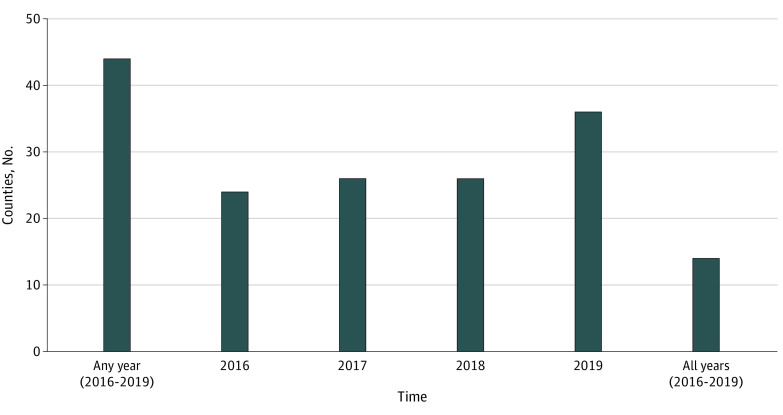
Number of Counties With at Least 1 Gun Violence Restraining Order by Year, 2016 to 2019

**Figure 3.**
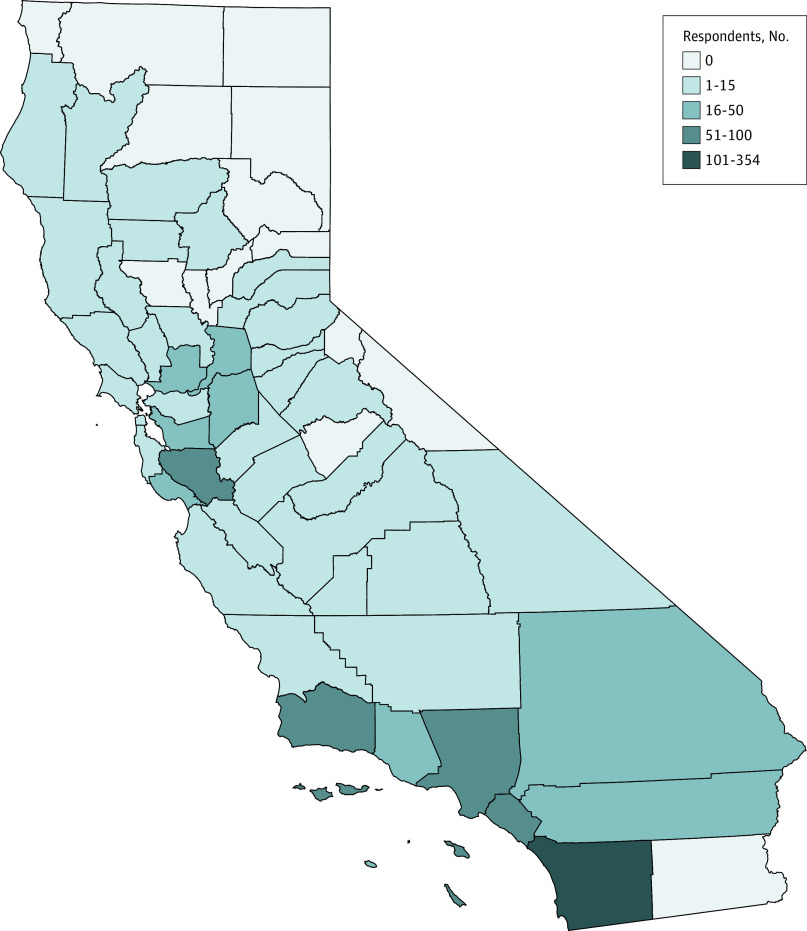
Number of Gun Violence Restraining Order Respondents in California Counties, 2016 to 2019

Overall, 44 of 58 counties (75.9%) issued at least 1 GVRO from 2016 to 2019, and 14 counties (24.1%) issued at least 1 in each year since the policy took effect (Figure 2). County-level spatial clustering in GVRO respondent counts was significant (observed Moran I, 0.18, mean [SD] Moran I from reference distribution, −0.01 (0.05); *z* value, 3.58; *P* = .004). The observed Moran I statistic (0.18) indicated a weak positive association between the number of respondents in any county and that of its neighbors. Counties in southern California as well as Santa Clara County most frequently used GVROs (eg, San Diego County, 354 respondents [32.9%]; Los Angeles County, 92 respondents [8.6%]; Orange County, 88 respondents [8.2%]; Santa Clara County, 88 respondents [8.2%]) (Figure 3). The count of respondents per county roughly matches population size. There was no significant spatial clustering in county rates of GVRO respondents per population (eTable 2 in the Supplement).

## Discussion

ERPO and risk warrant policies provide flexible, proactive, and individualized legal tools to temporarily prohibit possession and purchase of firearms and ammunition by individuals who pose a risk of harm to themselves or others but who do not meet existing prohibiting criteria. While both law enforcement and family or household members can petition for GVROs in California, petitioners in the first 4 years were overwhelmingly law enforcement officers.

GVROs were issued more often over time. However, rates of service decreased, and orders do not take effect until they are served. Prior research on restraining orders has reported high nonservice rates.^17,18^ Anecdotally, we understand reasons for nonservice include that the situation was resolved prior to service or that another prohibiting civil or criminal mechanism was used, rendering the GVRO unnecessary. We were unable to distinguish between unreported service and nonservice, both of which may vary over time, geographically, and by agency. Further exploration of court records and temporal and geographic variation could help clarify drivers of service rates and reporting within and between counties.

After 4 years of implementation, most counties had issued GVROs. San Diego County drove the increase, with more than 50 times the number of respondents in 2019 (267) than in 2016 (5). San Diego City Attorney Mara Elliott has repeatedly publicly endorsed GVROs, encouraged their use as a firearm violence prevention measure, and funded a team devoted to this effort.^13^ Her endorsement may help explain the disproportionate increase in use in San Diego, suggesting that local leadership may play a role in local policy use. San Diego has developed and begun implementing law enforcement training in other counties, which may to some extent account for the disproportionate use of GVROs in southern California compared with other areas of the state and explain the geographic clustering of counts of GVROs we observed.

Clustering of county counts of respondents was significant, but clustering of rates of respondents (per 100 000 residents) was not. We believe rates are unstable at this early stage because use is changing rapidly. Furthermore, these rates are potentially misleading because rates could vary, both absolutely and relatively, depending on choice of any of several plausible denominators (eg, a county’s total population, population of men, population of firearm owners, or population of firearm owners who are a danger to themselves or others).

Despite the increase in orders over time, GVRO uptake in California appears slow relative to other states. Compared with California’s 70 respondents in the first year, Oregon, with a population one-tenth of that of California, issued 42 orders in the first 6 months of policy implementation.^19^ Maryland, where more types of individuals are permitted to petition for ERPOs, issued approximately 445 orders in the first 11 months.^20^ Such discrepancies may owe in part to differences in policies, firearm culture, or more stringent firearm purchasing laws in California, which may result in fewer nonprohibited and at-risk firearm owners relative to other states. Comparative work and further investigation may help with understanding such differences; however, heterogeneity in states’ policies should be carefully considered in any multistate analysis.

Evaluations of risk warrant legislation (which predates ERPO laws) indicate that such policies may be primarily effective for suicide prevention.^8,9^ Estimating a number needed to treat for GVROs in California may be helpful for measuring the policy’s effectiveness and for making comparisons among states. Further research will allow understanding of the frequency with which GVROs in California are being used for prevention of self-directed vs other-directed harm.

Although uptake in California may have been slower than in other states, more than 1000 Californians were subject to GVROs. This suggests that the policy filled a gap in existing firearm violence prevention strategies, although these data do not allow us to measure the policy’s effects on violence prevention. Additionally, the rapid increase in respondents seen from 2018 to 2019 likely did not reflect a rapid increase in the underlying need for GVROs (ie, a sudden increase in the number of people who were a danger to themselves or others). Instead, the increase may reflect a growing awareness of GVROs, which could be an indication of an emergent upward trend in use as awareness, training, and media coverage continue.

These results may be useful to policy makers, law enforcement officers, county officials, and other stakeholders in California as well as those in states considering or enacting ERPO legislation. For example, our finding that few GVROs were issued in California before 2019 casts doubt on the recent suggestion that ERPOs in California and other states had major effects on suicide.^21^ Given that the findings in California may not be generalizable across the country, similar studies of early ERPO use in other states could be useful for comparison with these findings. These findings raise important questions for our continuing process and outcome research, which involves review of case court records, respondent criminal arrests and mortality, and interviews with key stakeholders. For example, why does uptake in California appear slower than in other states? Why are most petitioners law enforcement officers? What explains the variation in county-level use over time? What is the effectiveness of this intervention in preventing interpersonal and self-directed violence? Answering these questions may improve future application of ERPO laws.

### Limitations

This study has limitations. Law enforcement or court personnel must report GVROs to CA DOJ for archive in CARPOS, but manual data entry leaves room for error. The data files from CA DOJ contain information only on the last order issued per respondent. Therefore, we were unable to describe multiple orders per person or total number of orders issued. This limitation necessarily resulted in an undercount of emergency and ex parte orders, given that we expect that respondents whose last order was an order after hearing had a prior emergency or ex parte order. Furthermore, some of the increase over time may be attributable to classifying respondents according to their last order, but this is likely minimal considering that the 2019 data, which we received from CA DOJ in a separate and subsequent installment, indicated that only 37 respondents had an order in 2016 to 2018 and another in 2019.

Anecdotal evidence suggests that in some counties, civil or criminal domestic violence restraining orders or other prosecutions that may eventually result in a firearm prohibition are sought concurrently with GVRO petitions. We are unsure of how common this practice of seeking GVROs as a so-called backup to other means of prohibition is and whether it has become more common. Consequently, we cannot discern whether and to what extent this affected the increase in respondent counts over time. CARPOS does not contain information on other orders or prohibitions, but ongoing work will explore GVRO respondents’ co-occurring orders and other prohibitions.

Clustering of GVRO counts may reflect the clustering of counties by population in California rather than the clustering of GVRO use. Nonetheless, we believe that factors aside from county population, such as local champions and available resources, are driving varying county-level GVRO use. Additionally, because these data do not include denied petitions, our analysis may not cover the full scope of California’s GVRO use. Because these data do not contain information on risk, respondents’ health or criminal histories, targets of harm, and other case details, this analysis can only describe the early use of GVROs and cannot provide insight into the reasons for the policy’s use (eg, self-directed or other-directed harm) or the policy’s consequences.

## Conclusions

This study describes the early utilization of an ERPO law. Petitioners were overwhelmingly law enforcement officers; uptake has increased slowly from 2016 to 2018, with rapid increase in 2019; and local public champions may be driving particularly rapid uptake in some areas. The geographic and temporal variation in use suggests that ERPO uptake, implementation, or need may vary as a function of local, time-varying characteristics, such as awareness of the policy, protocols for implementation, willingness to petition for and issue ERPOs, and the use of other mechanisms for firearm relinquishment (eg, criminal protective orders). The results of this study may inform policy makers and other stakeholders in California and elsewhere involved with ERPO policy implementation, education, and outreach.

## Notes

Supplement. **eTable 1. **Variables Available in the California Restraining and Protective Order System (CARPOS), 2016-2019 **eTable 2. **Rates of Gun Violence Restraining Order (GVRO) Respondents per 100 000 Residents in California Counties, 2016-2019
