# Error in Data Management and Number of Study Respondents

**DOI:** 10.1001/jamanetworkopen.2021.20448

**Published:** 2021-07-07

In the Original Investigation titled “Assessment of Extreme Risk Protection Order Use in California From 2016 to 2019,”^1^ published on June 18, 2020, the authors identified an inadvertent data management error, which affected the number of Extreme Risk Protection Order respondents included in the study and the distribution of their characteristics. As a result, 18 respondents were not included in the final count who should have been, and 43 respondents had their last order misclassified. The correct number of total respondents is 1094, not 1076, and there are related corrected numbers in the Abstract, text, table, figures, and Supplement. None of the corrections affect the direction of any statistically significant or nonsignificant findings, interpretations, or conclusions. This article has been corrected.^1^
