# Supplementary material for: Assessment of Extreme Risk Protection Order Use in California From 2016 to 2019
Source: JAMA Netw Open. 2020 Jun 18;3(6):e207735. doi: 10.1001/jamanetworkopen.2020.7735 (PMC7303810; doi:10.1001/jamanetworkopen.2020.7735)
Supplement: Supplement. — eTable 1. Variables Available in the California Restraining and Protective Order System (CARPOS), 2016-2019 eTable 2. Rates of Gun Violence Restraining Order (GVRO) Respondents per 100 000 Residents in California Counties, 2016-2019 [file jamanetwopen-3-e207735-s001.pdf]

Supplementary Online Content

Pallin R, Schleimer JP, Pear VA, Wintemute GJ. Assessment of extreme risk protection order use in California from 2016 to 2019. *JAMA Netw Open*. 2020;3(6):e207735. doi:10.1001/jamanetworkopen.2020.7735

eTable 1. Variables Available in the California Restraining and Protective Order System (CARPOS), 2016-2019
eTable 2. Rates of Gun Violence Restraining Order (GVRO) Respondents per 100 000 Residents in California Counties, 2016-2019

This supplementary material has been provided by the authors to give readers additional information about their work.

© 2020 Pallin R et al. *JAMA Network Open.*

eTable 1. Variables Available in the California Restraining and Protective Order System (CARPOS), 2016-2019

Respondent name
Petitioner name
Additional respondent personal identifiers (FBI no., CII no., driver license no.)
Respondent date of birth
Respondent gender
Respondent race
Respondent height
Respondent weight
Respondent hair color
Respondent eye color
Order issue date
Order expiration date
Order type
Petitioner relationship to respondent (law enforcement or family)
Name of law enforcement agency (if law enforcement petitioned for the order)
Law enforcement agency identification number (ORI)
Agency case number(s)
Court case number
Court code
Respondent present in court (yes/no)
Respondent's current city
Respondent's current zip code
Name of person who served order
Service date
Service time
Agent ID (if law enforcement served the order)
Name of service agency (if law enforcement served the order)
Serving law enforcement agency identification number (ORI)
Record status at time of record retrieval (i.e., active, suspense, or history)

© 2020 Pallin R et al. *JAMA Network Open.*

eTable 2. Rates of Gun Violence Restraining Order Respondents per 100 000 Residents in California Counties, 2016-2019

County	Respondents per 100 000
Colusa	0.00
Lassen	0.00
Yuba	0.00
Del Norte	0.00
Mono	0.00
Modoc	0.00
Shasta	0.00
Imperial	0.00
Alpine	0.00
Siskiyou	0.00
Sutter	0.00
Mariposa	0.00
Sierra	0.00
Plumas	0.00
Tulare	0.05
El Dorado	0.13
Fresno	0.15
Madera	0.16
San Francisco	0.17
Humboldt	0.18
Merced	0.18
Kern	0.22
Los Angeles	0.23
Monterey	0.23
Alameda	0.26
Contra Costa	0.26
Stanislaus	0.27
Sonoma	0.30
Butte	0.33
San Mateo	0.39
Tehama	0.39
San Benito	0.41
San Bernardino	0.44
Riverside	0.45
Yolo	0.46

© 2020 Pallin R et al. *JAMA Network Open.*

Kings	0.50
Nevada	0.50
Napa	0.54
Calaveras	0.55
Placer	0.58
San Joaquin	0.67
Orange	0.69
Sacramento	0.74
San Luis Obispo	0.79
Mendocino	0.86
Ventura	0.97
Solano	1.07
Santa Clara	1.14
Marin	1.15
Inyo	1.39
Glenn	1.79
Tuolumne	1.84
Lake	1.95
Trinity	1.98
Amador	2.58
San Diego	2.66
Santa Barbara	3.36
Santa Cruz	4.01

Moran’s I = -0.03, p-value = 0.49.

Note: Respondents are assigned to the county in which their last order was issued.

© 2020 Pallin R et al. *JAMA Network Open.*
